# Biocompatibility and resorption pattern of newly developed hyaluronic acid hydrogel reinforced three-layer poly (lactide-co-glycolide) membrane: histologic observation in rabbit calvarial defect model

**DOI:** 10.1186/2055-7124-18-12

**Published:** 2014-09-24

**Authors:** Hoon You, Eun-Ung Lee, You-Kyoung Kim, Bum-Chul Kim, Jin-Young Park, Hyun-Chang Lim, Jung-Seok Lee, InSup Noh, Ui-Won Jung, Seong-Ho Choi

**Affiliations:** Department of Periodontology, Research Institute for Periodontal Regeneration, College of Dentistry, Yonsei University, Seoul, 120-752 Korea; Department of Chemical and Biomolecular Engineering, Seoul, 139-743 Korea; Convergence Institute of Biomedical Engineering and Biomaterials, Seoul National University of Science and Technology, Seoul, 139-743 Korea

**Keywords:** Hyaluronic acid, Hydrogel, Rabbit calvaria, Poly (lactide-co-glycolide), Biocompatibility

## Abstract

**Background:**

The aim of this study was to evaluate the biocompatibility and resorption pattern in three-layer poly (lactide-co-glycolide) (PLGA) membrane according to the concentrations of hyaluronic acid (HA) hydrogel in rabbit calvarial defect model. Four standardized circular defects with 8 mm diameter were created on the four rabbit calvarium. Three-layer PLGA membranes (5% and 10% HA gel) were used as the test groups, both collagen membrane and monolayer PLGA membrane as the control groups.

**Results:**

After sacrificing the animals after 4 and 8 weeks, block sections were harvested and histological observation was performed. Pus formation was observed in a site on the three-layer PLGA membranes (with 10% HA gel) of 4 weeks group and initial inflammatory responses were observed on the three-layer PLGA membrane group. However, when compared to both the monolayer PLGA membrane group and collagen membrane group, the HA gel-reinforced three-layer PLGA membrane showed improved cell occlusion and retention period, showing the formation of the capsule-like structure. There was no definite difference between the results of the membranes fabricated with either 5% or 10% HA hydrogel.

**Conclusion:**

The HA reinforced three-layer PGLA membrane was retained longer than control group and showed good property in cell occlusion. Future study is under process to improve the inflammatory response of the three layer PLGA membranes, which were observed in this study.

## Background

Numerous membranes have been developed by researchers in dental applications. Collagen membrane, as the most widely used resorbable membrane, usually have superior biocompatibility and tissue affinity than synthetic membrane due to its excellent biological properties [1, 2]. But its mechanical strength was rather lower than required in its applications in periodontal tissues. Furthermore its resorption rate has been reported to be unpredictable by the effects of enzymes and bacteria present in the periodontal tissue [3–7]. Recently, in order to overcome the limitations of the collagen membranes in their applications, the membranes fabricated by synthetic polymers such as poly (lactic acid) (PLA), poly (glycolic acid) (PGA), poly (lactic-co-glycolic acid) (PLGA) have been developed and their mechanical and resportion properties have been evaluated by many research groups [8, 9]. Not only in membranes, but in surgical supplies (sutures and fixation screw, etc.) and drugs they were also used and evaluated [10].

However, due to the hydrophobicity, stiffness and low tissue affinity of the reported PLGA membranes, there were difficulties in its clinical applications. And in some cases, inflammatory responses were reported during degradation of PLGA membrane. As examples, Von Arx et al., [11] by their histological study, reported that foreign body reaction occurred due to the degradation products of PLGA membranes. In particular, degradation of PLGA membrane induced a localized acidic environment. Therefore, complement for the problem was required [12].

To reduce the acidification issues, addition of biocompatible polymers to PLGA scaffolds have been tried to enhance their biocompatibility, one of which hyaluronic acid (HA) was used to increase the biocompatibility [13]. Because the PLGA has an ester molecular structure, hydrolysis occurs and acidification is induced. When hydrogen cations occur around HA, HA polymer reacts with the hydrogen cation. Then, hemiacetal ring remains and glucuronic acid residues are produced due to secondary decomposition. Finally, water and carbon dioxide are generated in the acidification of the PLGA. In short, HA was expected to work as a neutralizer of the acidification of PLGA.

In this study, to prevent local acidification of PLGA and to promote regeneration of lower hard tissue and upper soft tissue of the membrane, we designed HA reinforced three-layer PLGA membrane by applying the HA hydrogel in/on the PLGA disks in this study. In related studies, Lee et al. [14] reported that the PLGA/HA samples revealed faster cell adhesion and proliferation at *in vitro* experiments than those of PLGA and PLGA/HA. West et al. [15] also reported that HA metabolism played an important role in regulating angiogenesis because HA fragments induced the generation of new blood vessels [16]. Although many studies have been variously performed for the effects of HA on the functions of PLGA membrane [16], thus far the studies were only limited to either *in vitro* or few studies related to the effects of the HA concentrations.

The purpose of this study was to observe the biocompatibility and resorption pattern of PLGA membrane by histological comparative analyses by applying 10% (maximum titrating concentration) and 5% (medium concentration) of HA to the porous PLGA disks in rabbit calvarial defect model. To do this, the histological results of the samples implanted for 4 and 8 weeks were compared with after applying the collagen membrane, monolayer PLGA membrane, HA reinforced three-layer PLGA membrane in rabbit calvarial defects.

## Methods

### Animals

Four 16-week-old New Zealand white male rabbits, each weighing 3.0 ~ 3.5 kg, were used. Animal selection, management, preparation, and the surgical protocol followed the protocol that was approved by the Institutional Animal Care and Use Committee, Yonsei Medical Center, Seoul, Korea.

### Materials

#### Three-layer PLGA membrane

1. Manufacturing PLGA scaffold

**Figure 1:**
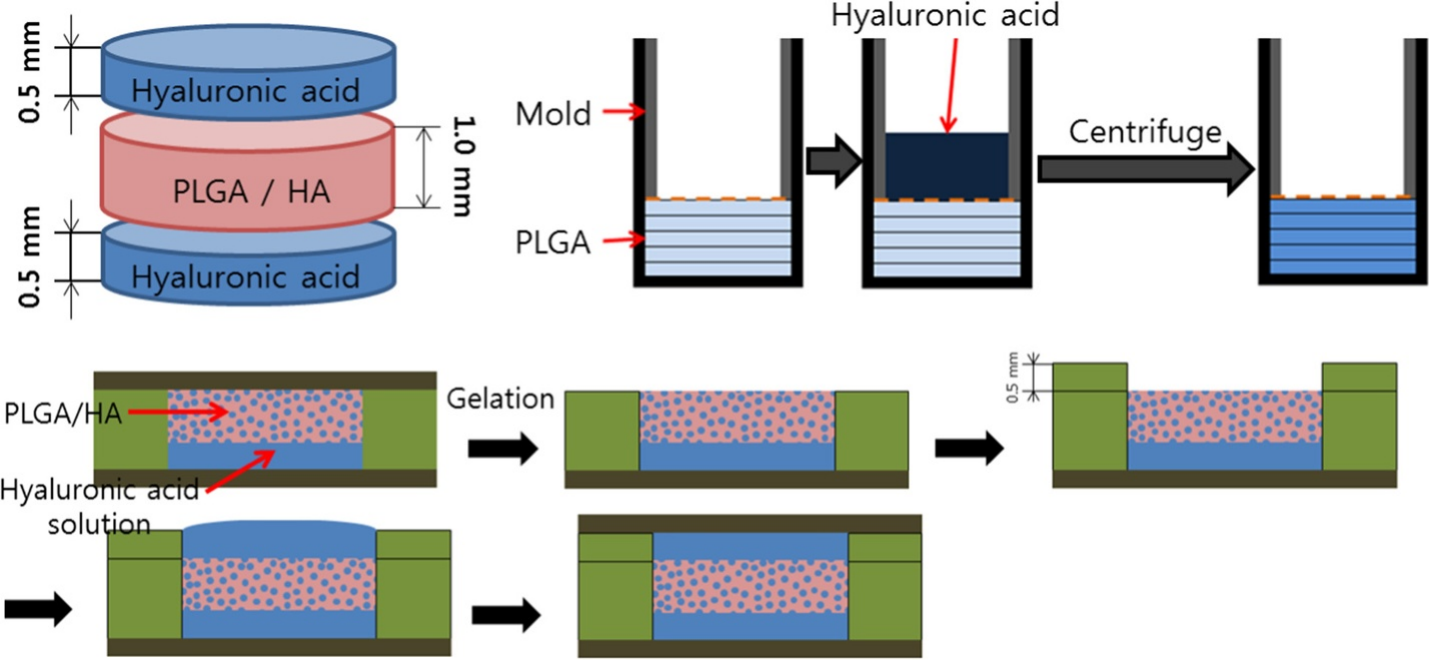
**Scheme of three-layer PLGA membrane.** First, centrifugation was performed to incorporate hyaluronic acid gel in a porous PLGA membrane. And hyaluronic acid hydrogel layers were attached on both sides of hylauronic acid incorporated PLGA membrane.

0.18 g of poly (lactic-co-glycolic acid) (PLGA; 65:35; Sigma-Aldrich Chemical Co., USA) was stirred 1 ml of dioxane for one day in order to prepare the completely dissolved PLGA polymer solution, and the prepared polymer solution was injected into a cylindrical shaped polyethylene (PE) mold with 10.0 mm diameter. Ammonium bicarbonate (AB; Sigma Aldrich Chemical Co.) particles, 355 ~ 500 μm diameter particle size, was mixed in the polymer solution. The prepared solution was rapidly cooled down by using liquid nitrogen. During freeze-drying performed for four days, dioxane was removed from the PLGA solution, thus obtaining a rod type sample.After removal of dioxane, the PLGA rod was cut uniformly in a disk with a thickness of 1.0 mm, and residual dioxane was completely removed again through lyophilizing of the samples for 24 hrs. The entire PLGA disks were immersed in the 4 liter of distilled water at 45°C and a porous PLGA scaffold was obtained by removing the AB particles with gasification in distilled water, three times at intervals of one hour. After drying the obtained porous PLGA disks under reduced pressure for 24 hrs, the sample was collected (5% HA gel; Figure 1).

2) Induction of HA hydrogel in the pores of the porous PLGA disks

Both 0.01 g of hyaluronic acid-acrylate polymer powder (HA-Ac) and 0.01 g of hyaluronic acid derivative polymer powder with phosphate functional group (HA-TCEP) were separately dissolved in 0.2 ml of PBS solution to create each precursor solution. In brief, HA-Ac has been synthesized by grafting acrylic acid to the hydroxyl groups of HA via 1-ethyl-3-(3-dimethylaminopropyl) carbodiimide (EDC) chemistry. Hyaluronic acid- tri(2-carboxyethyl)phosphine (HA-TCEP) was synthesized through similar mechanism by grafting TCEP to those of HA by using EDC chemistry, which was reported by one of authors in this paper [17].

3) Manufacturing three-layer PLGA membraneBoth 0.005 g of HA-Ac powder and 0.005 g of HA-TCEP powder were separately dissolved in 0.1 ml of PBS buffer solution to create each precursor solution. The prepared HA derivative solutions were mixed and then loaded on the top side of the PLGA disk in a Teflon mold (1 mm x 1 mm). Gelation was proceeded in 4°C for 1 hr, thus obtaining a two-layered scaffold. This same process was performed on the other side of the two-layered scaffold, thus obtaining a three-layer PLGA membrane (Figure 1). 10% HA derivative solution was employed for manufacturing of 10% 3-layer PLGA membrane by following the same protocol as described above.

#### Collagen membrane

A non cross-linked collagen membrane with type I and III porcine collagen (Bio-Gide®, Geistlich-Pharma, Wolhusen, Switzerland) were employed as a control and used as supplied. It had a smooth, compact upper layer and a dense porous lower layer.

#### Monolayer PLGA membrane

A porous PLGA scaffold was manufactured in a membrane type with 2 mm thickness. The monolayer PLGA membrane group was employed as a control for comparison with the three-layer PGLA membrane group (2.0 mm thickness).

### Study design

While both collagen and monolayer PLGA membrane groups were used as control groups, the three-layer PLGA membrane groups (5% and 10% HA gel) were employed for experimental groups. Bone graft material was not used to exclude possible influence of graft material on the study. Each group was sacrificed at 4 and 8 weeks after surgery. Two rabbits were assigned to each group, and total four rabbits were used for experiments.

### Surgical Protocol

**Figure 2:**
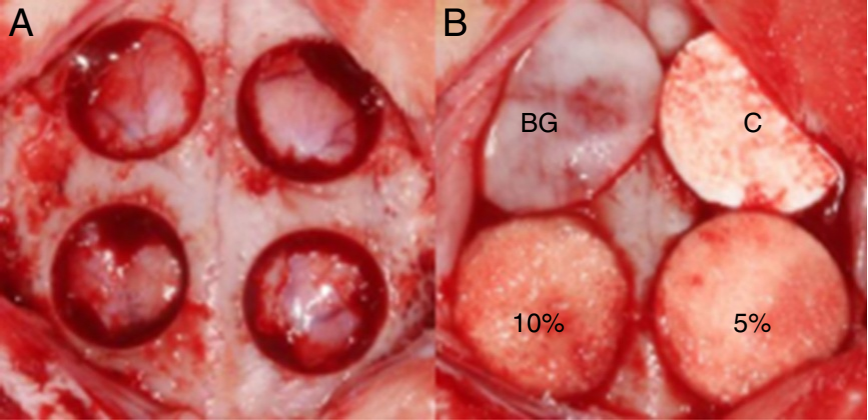
**Rabbit calvarial defect model. (A)** Critical size calvarial defects (8 mm diameter) were created, two defects on each side of the sagittal suture. **(B)** Four defects were covered with different membranes, where BG: Bio-Gide^®^; **C**: Monolayer PLGA membrane; 5%: 3-layer PLGA membrane (5% HA gel); and 10%: 3-layer PLGA membrane (10% HA gel).

Hair removal and disinfection procedures were carried out with povidine iodine under general anesthesia with ketamine hydrochloride and xylazine. An incision was made in the sagittal plane across the cranium, and a full thickness flap was lifted to expose the calvarial bone under infiltration anesthesia with 2% lidocaine. Four bone defects were formed around sagittal plane with 8 mm diameter trephine bur under saline irrigation (Figure 2A). Collagen membrane, monolayer PLGA membrane and three-layer PLGA membrane (5% and 10% HA gel) were applied on prepared four bone defects (Figure 2B). After the barrier was positioned evenly, periosteum suture using resorbable suture material was performed to fix the membrane and skin closure with mono-filament nylon suture material was done.

After 4 and 8 weeks of surgery, the experimental animals were sacrificed with phenobarbital (100 mg/kg) by intravenous injection. Samples were fixed with 10% neutral formalin for 10 days and demineralized with 5% nitric acid for 5 days. Then the demineralized tissues were embedded in paraffin. Serial sections were performed with 7 μm thickness, stained with hematoxylin–eosin (H–E). All the animal experiments followed the animal guideline of Yonsei University Medical School (Project number: 2013–0042).

### Evaluation methods

#### Clinical observations

During the procedures of dressing, stitch-out and 4 and 8 weeks of surgery, possible complications such as inflammation and abnormal reactions on the surgical sites were observed.

#### Histological observations

At the time points of 4 and 8 weeks after surgery, the appearance of new bone formation, membrane resorption, angiogenesis, cells of inflammation, and infiltration of external organisms were observed.

## Results

### Clinical observations

Inflammatory response was observed on a 10% HA hydrogel reinforced three-layer PLGA membrane in one of 4 weeks group during the healing period. There was dome-shaped elevation with pus formation at the time of sacrifice. Remarkably specific findings were not observed on the other samples.

### Histologic observations

#### Collagen membrane (Bio-Gide®)

**Figure 3:**
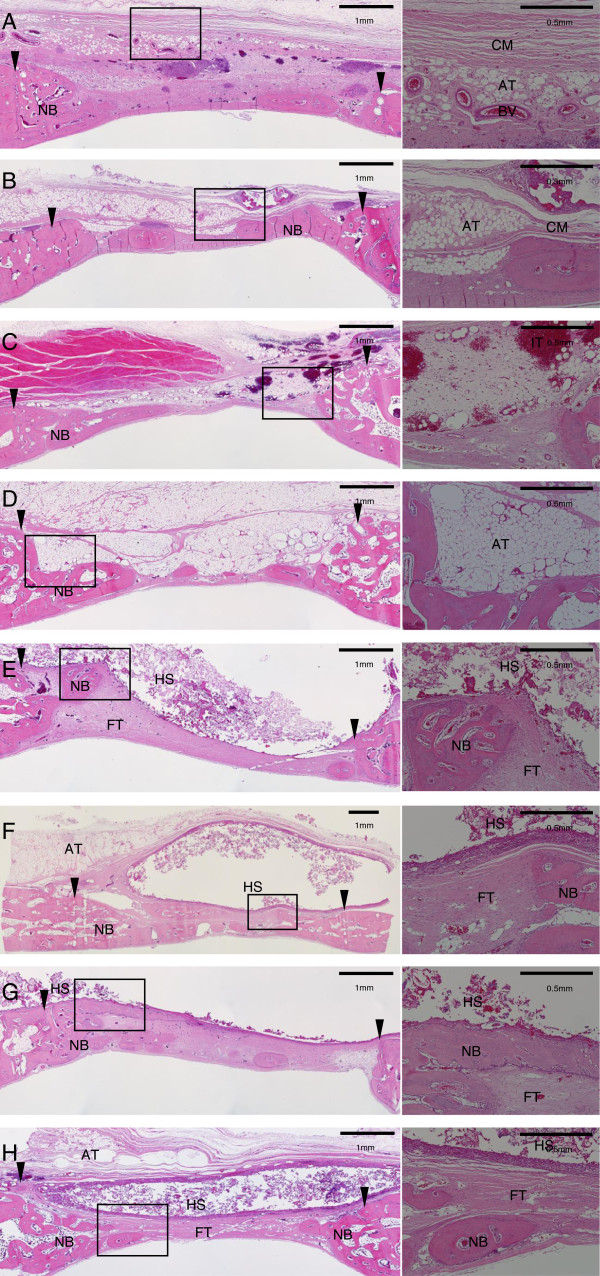
**Histological view of rabbit calvarial defect. (A)** Collagen membrane (Bio-Gide^®^) group at 4 weeks, and **(B)** 8 weeks. **(C)** Monolayer PLGA membrane group at 4 weeks, and **(D)** 8 weeks. **(E)** 3-layer PLGA (5% HA gel) membrane group at 4 weeks, and **(F)** 8 weeks. **(G)** 3-layer PLGA (10% HA gel) membrane group at 4 weeks, and **(H)** 8 weeks. H-E stain. (Left - ×40, Right - ×100) (arrowhead: defect margin, BV: blood vessel, NB: new bone, CM: collagen membrane, AT: adipose tissue, IT: inflamed tissue, FT: fibrous tissue, HS: hydrogel scaffold).

In 4 weeks group of collagen membrane, there were dense and neat collagen fibers that run parallel to the bone defect. In addition, it seemed that angiogenesis had been made actively (Figure 3A). However, there were penetrations of adipose tissue both in 4 and 8 weeks group. And, there was premature collapse of the membrane, either. Consequentially, declined new bone formation was observed in the area of collapsed membrane. In particular, more adipose tissue infiltration could be observed in 8 weeks group than 4 weeks group (Figure 3B).

#### Monolayer PLGA membrane

Monolayer PLGA membrane had been collapsed when observed at 4 weeks, and the inflamed tissue was observed (Figure 3C). In 8 weeks group, external organism (mainly in adipose tissue) with discontinuity of the membrane due to its premature resorption was widely observed. There was thick layer containing adipose tissue between bone defect and membrane, and definitively decreased new bone formation was observed in that area compared with those of the other groups (Figure 3D).

#### Three-layer PLGA membrane (5% HA gel)

New bone growth into the defect area with upward and downward of marginal bone resorption was shown in 4 weeks group. It could be observed that fibrous tissue had been regularly arranged inside the new bone. There was hydrogel scaffold above the fibrous tissue. In the high-magnification slide, proliferation and migration of vascular tissue had begun, and new bone took place more actively in one side in which there had been relatively well-maintained hydrogel scaffold (Figure 3E).The three-layer PLGA membrane was maintained to prevent the infiltration of outer adipose tissue in the 8 weeks group. Scattered hydrogel was maintained in shape of the capsule, and inflammatory cells such as monocyte took their position on inner layer, fibrous connective tissue on outer layer of the capsule. New bone had been actively formed beneath of the capsule, and it could be observed that most width of bone defect had been replaced with new bone. In the high-magnification slide, osteoblasts had been actively formed around new bone (Figure 3F).

#### Three-layer PLGA membrane (10% HA gel)

With the exception of small amount residue of hydrogel, most of the hydrogel scaffold and membrane were separated from bone defect site in 4 weeks group. There was pus on that site due to inflammatory response. However except for the right defect margin of Figure 3G, adipose tissue infiltration was not observed, and the top of the defect portion was filled with new bone which was beneath of HA hydrogel residue. In the high-magnification slide, new bone was formed actively on the one side where HA hydrogel had remained, whereas it was scarcely formed on the other side where HA hydrogel had failed due to infiltration of adipose tissue (Figure 3G).Such as the former 5% HA hydrogel reinforced three-layer PLGA membrane group, HA hydrogel scaffold was observed as capsule shaped in the 8 weeks group, and new bone formation could be seen in about 60% width of the bone defect below the HA hydrogel scaffold. Penetration of adipose tissue into cleft of the membrane could be observed in left side of Figure 3H, but downward penetration of it through the HA hydrogel scaffold was not observed (Figure 3H).

## Discussion

HA have been used in various fields of medical applications such as drug carrier and biological materials [17, 18]. Its biological advantages have been reported such as bioresorbable and less immune responsible materials. In addition, it has properties of osteoconduction, and induces angiogenesis [19].

However, inflammatory response accompanied by pus formation was observed in one of the three-layer PLGA membrane (10% HA gel) in 4 weeks group, and inflammatory cells on inner layer of the capsule were infiltrated in three-layer PLGA membrane in this study. Von Arx et al. [11] reported that fibrous capsulation and infiltration of inflammatory cells were observed in PLGA membrane after 2 weeks.

The first reason of inflammation may be due to the fact that the degradation products of PLGA might act as foreign body. Schmidmaier [20] and Dimitriou [21] et al., reported that foreign body reaction was caused by degradation of PLGA membrane. In this regards, the foreign body reaction seemed to depend on diverse factors such as polymer type, size and cross-linking techniques and manufacturing processing. Second, it can be assumed that incomplete removal of dioxane which was used as the solvent for the fabrication of PLGA scaffolds resulted in inflammatory response. Ammonium bicarbonate which was added to form the pores of the porous PLGA might be also one of potential infection sources.

Despite histologically observed inflammatory response, new bone was formed beneath the three-layer PLGA membrane. It seems that HA might induce rapid adhesion and proliferation of osteoblast. Similar results are observed in many different studies. As examples, Marinucci [22] et al. reported that HA stimulated proliferation of osteoblast through *in vitro* study, and Park [9] et al. reported that more bone formation was observed in the HA applied PLGA membrane than in either PLGA or collagen membrane in white rat calvarial defects.

Premature degradation of the collagen membrane partially occurred at 4 weeks, but overall contour was sustained. More extensive premature breakdown of membrane accompanied by sporadic proliferation of inflammatory cell also occurred in monolayer PLGA membrane group. Jung [8] et al. reported that the result of PLGA membrane degradation was worse than the collagen membrane because of its accelerated breakdown. It was considered that monolayer PLGA membrane used in this study was behind in biocompatibility and retention period than collagen membrane.

On the other hand, in the three-layer PLGA membrane, HA hydrogel was formed in fibrous capsule-like structure supporting the whole contour of membrane and preventing infiltration of external organism during the period of observation. In other words, the HA hydrogel reinforced three-layer PLGA membrane was considered that it showed improved retention period and cell occlusion. However, in comparison 5% HA hydrogel with 10% HA hydrogel, no definite difference was observed in biocompatibility and retention period of the membrane. Afterwards, further experimental observations need to be conducted by revision of study design, including expansion of experimental populations, modification of proper concentration of HA hydrogel, fabrication of 3-layer scaffolds and long-term observation of healing group.

## Conclusion

This study investigated the probability of applications of the three-layer PLGA membrane to the membrane by using different concentrations of HA hydrogel in/on the PLGA membrane. Different level of inflammatory response was observed in the newly developed three-layer PLGA membrane as well as the good property in cell occlusion. From the results, the HA hydrogel reinforced three-layer PLGA membrane had longer retention period than collagen membrane and monolayer PLGA membrane had in rabbit calvarial defects. Further study is under progress to improve the inflammatory response observed in this study.

## Availability of supporting data

The data sets supporting the results of this article are included within the article.
